# Prevalence of Complementary and Alternative Medicine Use and Its Associated Factors among Iranian Diabetic Patients: A Cross-Sectional Study

**DOI:** 10.1016/j.curtheres.2024.100746

**Published:** 2024-03-21

**Authors:** Fereshteh Ghorat, Seyed Hamdollah Mosavat, Samaneh Hadigheh, Seyed Amin Kouhpayeh, Mohammad Mehdi Naghizadeh, Ali Akbar Rashidi, Mohammad Hashem Hashempur

**Affiliations:** 1 Non-Communicable Diseases Research Center, Sabzevar University of Medical Sciences, Sabzevar, Iran; 2 Research Center for Traditional Medicine and History of Medicine, Department of Persian Medicine, School of Medicine, Shiraz University of Medical Sciences, Shiraz, Iran; 3 Student Research Committee, Babol University of Medical Sciences, Babol, Iran; 4 Non-Communicable Diseases Research Center, Fasa University of Medical Sciences, Fasa, Iran

**Keywords:** Complementary and alternative medicine, Diabetes mellitus, Integrative medicine, Iran, Medicinal herbs, Quality of life, Traditional Persian medicine

## Abstract

**Objective:**

This cross-sectional study aimed to assess the prevalence of complementary and alternative medicine (CAM) use and its associated factors among diabetic patients in Fasa, a city in southern Iran.

**Methods:**

Data were collected from diabetic patients who visited the endocrinology clinics at Fasa University of Medical Sciences. A structured questionnaire was administered to gather information on CAM use, including the types of CAM modalities used, and reasons for use. The patient's demographic and clinical characteristics, such as age, gender, duration of diabetes, glycosylated hemoglobin (HbA1c) levels, and quality of life (QoL) were also recorded. Descriptive statistics were used to determine the prevalence of CAM use, while logistic regression analysis was employed to identify factors associated with CAM use.

**Results:**

A total of 376 diabetic patients participated in the study, with more than 89% reporting CAM use within the past year. Herbal preparations were the most commonly used type of CAM, with a prevalence rate of 99.4%. Factors associated with CAM use included patients’ psychological health, attitude towards the safety of CAM, belief in the synergistic effects of combining routine medications with CAM, and previous positive experiences with CAM.

**Conclusion:**

The high prevalence of CAM use highlights the importance of considering it in diabetes management and the need for healthcare professionals’ engagement in open discussions with patients about their CAM practices. Understanding the factors influencing CAM use can inform healthcare providers and policymakers in developing appropriate strategies for integrating CAM approaches into conventional diabetes care.

## Introduction

Complementary and alternative medicine (CAM) refers to a diverse range of health practices, approaches, knowledge, and beliefs that incorporate natural remedies, spiritual therapies, manual techniques, and exercises.^1^ It has been recognized by the World Health Organization (WHO) as a means to maintain well-being and treat, diagnose, or prevent diseases.^2^ Popular systems of CAM include Traditional Chinese Medicine, Indian Ayurveda Medicine, homeopathy, traditional Persian medicine, and chiropractic, which have gained significant popularity worldwide.^3^

Studies have shown that a considerable portion of the population in developing countries, such as African nations, rely on traditional medicine for primary healthcare, with usage rates reaching up to 80%.^4^, ^5^, ^6^ However, it is important to note that the use of CAM is not exclusive to developing countries, as research has also highlighted its prevalence among individuals in developed countries.^7^^,^^8^ Factors contributing to the increased use of CAM in developing countries include its accessibility, affordability, and concerns regarding the adverse effects of chemical drugs.^9^^,^^10^ In developed countries, individuals are increasingly questioning the approaches and assumptions of mainstream medicine and greater access to health information has led to a rise in alternative healthcare approaches.^11^^,^^12^

Despite the long-standing existence and widespread use of CAM throughout history, it has not received official recognition in many countries.^13^ The lack of convincing safety and efficacy data has been a significant barrier to its acceptance.^14^ To address this gap, we conducted a cross-sectional study aimed at evaluating the prevalence of CAM use, types of CAM use, and associated factors in patients with type 2 diabetes mellitus (T2DM).

T2DM is a complex and multifaceted chronic disease with a rising global prevalence, posing significant challenges to public health worldwide.^15^ The selection of T2DM as the focus of our research stems from compelling epidemiological and clinical considerations that underscore its profound impact on individuals and healthcare systems. The escalating prevalence of T2DM, both in developed and developing nations, emphasizes the urgency of understanding the factors influencing its management and patient outcomes.^16^

Epidemiologically, T2DM represents a considerable burden on healthcare systems, with a global estimate of 463 million people affected in 2019, and projections suggesting a rise to 700 million by 2045.^17^ This escalating prevalence necessitates a comprehensive exploration of the disease's nuances, especially in diverse populations, to inform tailored interventions and improve overall health outcomes.

Clinical considerations further underscore the significance of T2DM as a study pathology. Beyond its sheer prevalence, T2DM is characterized by a spectrum of complications, including cardiovascular disease, nephropathy, and retinopathy, leading to a substantial reduction in quality of life (QoL) and increased mortality.^18^ Understanding the intricacies of T2DM management becomes paramount, not only to alleviate the burden on healthcare systems but, more importantly, to enhance the well-being of individuals grappling with this condition.

Previous studies have delved into various aspects of T2DM, providing valuable insights into its epidemiology and clinical manifestations. Cuadros et al.,^19^ conducted a comprehensive population-based study highlighting the regional variations in T2DM prevalence, shedding light on potential geographical determinants. Moreover, Arnold et al.,^18^ delved into the intricate relationship between T2DM and cardiovascular outcomes, emphasizing the need for a holistic approach to patient care.

A seminal study by Khodakarami et al.,^20^ investigated the prevalence of diabetes in a representative Iranian population, offering a valuable epidemiological perspective. Their findings indicated a notable rise in the prevalence of diabetes over the past decade, underscoring the relevance of our current research in the Iranian context. Moreover, Mohseni et al.,^21^ delved into the intricacies of diabetes management, emphasizing the challenges faced by individuals in navigating the complexities of their treatment regimens. These studies collectively underscore the pressing need for a nuanced understanding of diabetes in the Iranian population, taking into account both epidemiological trends and the intricacies of patient care.

Iran, with its unique sociodemographic landscape, exhibits certain epidemiological peculiarities that differentiate its population from those of other countries. Notably, studies such as Najafipour et al.^22^ have highlighted distinct trends in the prevalence and incidence of T2DM within the Iranian population. Factors such as genetic predispositions, dietary habits, and lifestyle choices contribute to the nuanced epidemiological profile of diabetes in Iran. Furthermore, Khamseh et al.,^23^ delved into the regional variations in diabetes prevalence, emphasizing the importance of considering geographical factors in understanding the disease burden. The prevalence of risk factors, including sedentary lifestyles and dietary patterns, may vary in Iran compared to other nations, necessitating a tailored approach to diabetes research and care.

Against this backdrop, our study aims to contribute to the existing body of knowledge by examining factors influencing the utilization of CAM among individuals with T2DM in the Iranian context. By building upon the foundation laid by previous research, we endeavor to unravel unique patterns and determinants that may influence CAM usage, ultimately informing patient-centered care strategies in the management of T2DM.

## Methods and Material

### Study Design and Sample

This descriptive and analytic cross-sectional study was conducted on a convenience sample of 376 patients who sought healthcare at outpatient academic endocrinology clinics affiliated with Fasa University of Medical Sciences (FUMS). The study received approval from the Local Medical Ethics Committee of FUMS (ID 94016).

Participants in this study were individuals diagnosed with T2DM. Inclusion criteria comprised adults aged 18 years and above, with a confirmed diagnosis of T2DM based on criteria suggested by the American Diabetes Association.^24^

Exclusion criteria included individuals with other types of diabetes (e.g., type 1 diabetes) and those with significant cognitive impairment hindering questionnaire completion.

### Data Collection

In our study, the invitation to participate was extended by 3 members of the medical staff involved in endocrinology clinics. Trained research personnel administered the questionnaires to the participants. These individuals were not directly involved in the clinical care of the patients to avoid any potential bias. Before their participation, all patients were provided with detailed information about the study, and written informed consent was obtained from each participant. The informed consent process ensured that participants were fully aware of the study's purpose, procedures, potential risks, and benefits before voluntarily agreeing to take part in the research.

A self-administered, semi-structured questionnaire was used to collect data from diabetic patients aged 18 years and older who were on conventional medication. This questionnaire has been used in 2 studies that were similar to ours.^12^^,^^25^ In the initial stage, various demographic variables were examined, including age, gender, marital status, level of education, place of residence, type of diabetes medication, presence or absence of blood lipid disorders, and duration of illness. Subsequently, inquiries were made regarding the frequency of visits to the diabetes clinic in the past year, regular at-home blood glucose monitoring, participation in diabetes education classes, and the occurrence of diabetes-related complications.

To ensure clarity and consistency in data collection, the concept of CAM was explicitly explained to all participants before inquiries regarding its usage. Recognizing the potential variability in patients’ understanding of diverse CAM modalities, our research team took proactive measures to provide a comprehensive explanation. Participants were briefed on the inclusive nature of CAM, encompassing various unconventional therapeutic approaches beyond mainstream medical interventions. This preface aimed to enhance participants’ awareness, enabling them to discern and articulate their engagement with CAM accurately. After this step, patients were questioned about the use of herbal or traditional remedies. In our study, the questions about the use of CAM focused on the participants’ use in the past year. Specifically, we inquired about their current utilization of CAM therapies in managing their diabetes. The intention was to capture the most up-to-date information regarding CAM usage during the period of the study. If the response was affirmative, subsequent questions were asked regarding their communication with their physicians regarding the use of traditional therapies, the reasons for opting for such treatments (e.g., lipid and blood glucose control, general well-being), and the types of CAM modalities employed, such as herbal medicine, cupping, leech therapy, acupuncture, etc. Patients who utilized herbal remedies (e.g., thyme, mint, watermelon, colocynth) would specify their names. In this context, patients were also asked about their familiarity with CAM, including physicians and medical staff, family and friends, media, books, and herbalists. If patients had made modifications in their usage of conventional medications due to the utilization of traditional remedies, they were encouraged to share their experiences.

In the subsequent part of the interview, the patients were presented with a quality-of-life questionnaire. The assessment of participants’ QoL was conducted using the World Health Organization Quality of Life (WHOQOL-BREF) instrument.^26^ The WHOQOL-BREF demonstrates satisfactory psychometric characteristics, making it a suitable instrument for evaluating the QoL at the domain level among adults in the Iranian population.^27^ The WHOQOL-BREF comprises various domains, including physical health, psychological well-being, social relationships, and environmental factors. The assessment of participants’ QoL utilized the WHOQOL-BREF questionnaire, which encompasses 26 items distributed across 4 domains: physical health (7 items), psychological health (6 items), social relationships (3 items), and environment (8 items). Additionally, 2 items gauge the overall QoL and general health. Participants responded on a 5-point Likert scale (ranging from 1 for a low score to 5 for a high score) for each item, generating raw item scores. The mean score for each domain was then computed, resulting in a mean score per domain within the range of 4 to 20. To facilitate comparability with the original WHOQOL-100 scores, each mean domain score was multiplied by 4. The transformed domain scores, now on a scaled range, continue to reflect the participants’ perceived QoL. The scaled scores for each domain fall between 4 and 80 after transformation. A higher scaled score in each domain signifies an elevated QoL in that specific dimension. For example, a higher scaled score in the psychological health domain indicates a more positive perception of psychological well-being.

Following this section, the patient's weight and height were measured and recorded. Additionally, laboratory tests such as HbA1c which the patients had brought with them during their clinic visits were documented. If complete or up-to-date test results were not available, they were conducted free of charge for the patients.

### Objective Measurements

Objective measurements, including weight, height, blood pressure, and glycosylated hemoglobin (HbA1c), were obtained from all participants as basic characteristics.

### Statistical Analysis

Descriptive statistics such as means and proportions were used to summarize the data. Logistic regression, chi-square tests, and independent t-tests were employed for further analysis. *P*-values less than 0.05 were considered statistically significant. The Statistical Package for the Social Sciences version 15.0 (SPSS Inc., Chicago, Illinois) software was used for all statistical analyses.

## Results

### Socio-demographic Information

A total of 399 patients were initially invited to participate in the study. Among them, 376 patients willingly agreed to take part. Before the study, more than 89% of the patients had used CAM. The analysis of socio-demographic data revealed no significant differences in the rate of CAM usage based on sex, marital status, educational level, and urban or rural residency (all *P* > 0.05) (Table 1).

**Table 1:** Socio-demographic data of the participants (CAM users versus nonusers).

		CAM use				*P*-value	Odds ratio	95% Confidence intervals	
		Nonusers (n = 40)		Users (n = 336)					
		Count	%	Count	%			Lower	Upper
Sex	Female	33	10.7%	276	89.3%	0.956			
	Male	7	10.4%	60	89.6%		1.025	0.433	2.427
Marital status	Single	1	12.5%	7	87.5%	0.928			
	Married	31	10.3%	269	89.7%		1.240	0.148	10.411
	Other	8	11.8%	60	88.2%		1.071	0.116	9.879
Education	Uneducated	14	9.9%	128	90.1%	0.570			
	Up to diploma	25	11.7%	188	88.3%		0.823	0.412	1.643
	Academic	1	4.8%	20	95.2%		2.188	0.273	17.559
Residency	Urban	28	11.4%	217	88.6%	0.497			
	Rural	12	9.2%	119	90.8%		1.280	0.628	2.609
Age (mean years ± SD)		55.58 ± 12.12		55.8 ± 10.37		0.9	1.002	0.971	1.034

CAM = complementary and alternative medicine; DM = diabetes mellitus; SD = standard deviation.

### Diabetes Specific Information

Totally, 229 patients (60.9%) also had dyslipidemia. The average duration of diabetes in the participants was approximately 8 years. There were no significant differences in the duration of diabetes, number of clinic visits, self-monitoring of blood glucose, and participation in diabetes education programs between CAM users and nonusers (all *P* > 0.05). About 58% of patients reported diabetes-related complications such as retinopathy and neuropathy. No significant differences in CAM usage were observed based on body mass index, HbA1c level (all *P* > 0.05). Details of the basic clinical characteristics of the participants (CAM users versus nonusers) in each group are shown in Table 2.

**Table 2:** Basic clinical characteristics of the participants (CAM users versus nonusers).

	CAM use				*P*-value	Odds ratio	95% Confidence intervals	
	Nonusers (n = 40)		Users (n = 336)					
	Mean ± SD		Mean ± SD				Lower	Upper
Duration of DM (month)	98.63 ± 81.49		100.4 ± 75.6		0.889	1	0.996	1.005
Visit count	5.65 ± 3.25		6.5 ± 4.44		0.241	1.054	0.965	1.15
BMI (kg/m²)	27.35 ± 4.25		27.54 ± 4.46		0.803	1.01	0.935	1.091
Weight (kg)	69.78 ± 11.47		70.31 ± 12.4		0.795	1.004	0.977	1.031
SBP (mmHg)	120.51 ± 6.37		117.84 ± 26.81		0.86	0.995	0.943	1.05
HbA1c (%)	7.84 ± 1.56		7.65 ± 1.83		0.555	0.945	0.785	1.138
Use of Insulin No Yes	Count 28	% 10.2%	Count 247	% 89.8%	0.636	0.841	1.72	0.410
	12	11.9%	89	88.1%				
Use of OHA No Yes	Count 2	% 6.3 %	Count 30	% 93.8%	0.400	0.536	2.33	0.123
	38	11.0%	306	89.0%				
Complication of DM No Yes	Count 18	% 8.3%	Count 200	% 91.7%	0.079	0.556	0.288	1.076
	22	13.9%	136	81.6%				
Regular blood glucose Monitoring No Yes	Count 23	% 9.9%	Count 209	% 90.1%	0.563	0.822	1.598	0..423
	17	11.8%	127	88.2%				

CAM = complementary and alternative medicine; DM: diabetes mellitus; BMI = body mass index; SBP = systolic blood pressure; HbA1c = glycated hemoglobin; OHA = oral hypoglycemic agents.

### Quality of Life-Related Data

As shown in Table 3, among the quality-of-life domains, only the psychological health domain showed a significant association with the rate of CAM usage (OR = 1.027, 95% CI, 1.001–1.056). This indicates that individuals with a higher level of psychological health were more likely to use CAM. No significant associations were found between CAM usage and other QoL domains (physical health, social relationships, environmental health, overall QoL, and overall health status).

**Table 3:** Association between CAM use and quality of life domains among studied diabetic patients.

Quality of life domains	CAM Use		*P*-value	Odds ratio	95% Confidence intervals	
	Nonusers (n = 40)	Users (n = 336)				
	Mean ± SD	Mean ± SD			Lower	Upper
Physical health	50.00 ± 24.95	46.70 ± 25.43	0.437	0.995	0.982	1.008
Psychological health	51.04 ± 12.18	55.03 ± 12.13	0.050	1.027	1.001	1.056
Social relationships	47.92 ± 29.28	53.57 ± 28.42	0.237	1.007	0.996	1.018
Environmental health	45.70 ± 12.73	46.37 ± 13.10	0.759	1.004	0.979	1.029
Overall QoL	54.38 ± 32.47	45.46 ± 30.72	0.088	0.99	0.979	1.001
Overall health status	39.38 ± 37.08	37.35 ± 33.90	0.723	0.998	0.989	1.008

CAM = complementary and alternative medicine; QoL = quality of life.

### CAM Use Pattern in the Patients

Herbal preparations were the most common type of CAM used by patients (99.4%). Among CAM users, 334 patients used herbal remedies, while 10 patients (2.9%) used other types of CAM such as cupping and leech therapy. The primary source of recommendation for CAM use was family members and friends (86.6%). A large majority of patients (83.3%) used herbal remedies for different diseases. Notably, more than 90% of CAM users did not change their routine diabetes medication while using herbal preparations. Only 20.8% of patients informed their physicians about their use of herbal drugs. A detailed description of CAM use patterns in the patients is shown in Table 4.

**Table 4:** The pattern of use in CAM users.

	No.	%
Type of CAM*		
Herbal preparations	334	99.4
Cupping	8	2.4
Leech therapy	2	0.6
Recommended by*		
Health care providers	36	10.7
Family members, and friends	291	86.6
Media, books, and magazines	38	11.3
Herbal markets	62	18.5
**Alteration in routine medication of DM**		
Stopping of medication	10	3
Alteration in time of medication	1	0.3
Decreasing of medication dose	19	5.7
No change	306	91

CAM = complementary and alternative medicine; DM = diabetes mellitus.

⁎ The cumulative total of values could exceed 100% due to the allowance for patients to select multiple options.

In this study, the utilization of medicinal plants by individuals with diabetes was investigated. Patients not only sought these remedies for blood glucose control but also potential improvements in various domains such as lipid profiles, neurological health, cardiovascular function, and general well-being. The range of herbal medications employed was extensive, and thus, we present only a few of the most prevalent ones. Some instances were limited to the consumption by 2 to 3 individuals, rendering them inconclusive for meaningful analysis.

**Figure 1:**
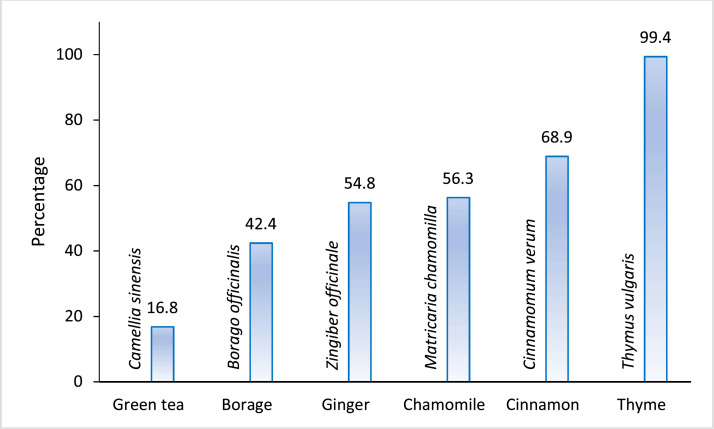
Prevalence of various medicinal plants use among CAM users.

A total of 332 patients reported the usage of thyme. Cinnamon exhibited a noteworthy prevalence among the participants. Chamomile, green tea, borage, and fenugreek followed in subsequent rankings, as depicted in Figure 1.

**Figure 2:**
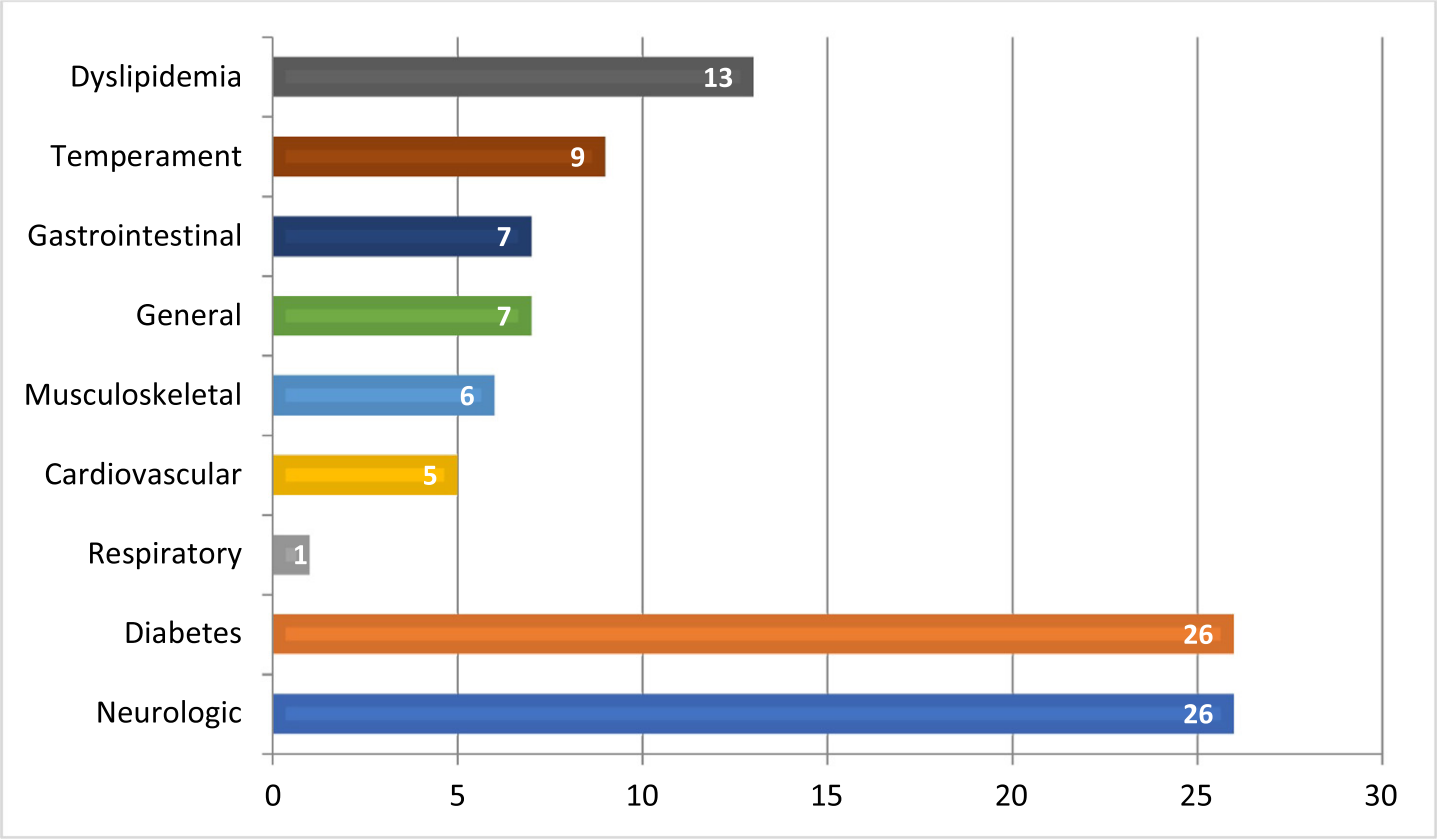
Motivations for herbal medicine usage among CAM users.

In this study, we conducted inquiries regarding the motivations behind the utilization of different herbal medicines among the patient cohort. The results revealed that the primary reasons for usage were blood glucose control and nervous system relaxation, as reported by a majority of participants. Additionally, secondary reasons included the management of lipid disorders, enhancement of overall well-being, resolution of digestive system issues, improvement of musculoskeletal health, promotion of cardiovascular health, reinforcement of immune function, and support for respiratory health. These findings are visually represented in the accompanying Figure 2.

Table 5 provides a detailed breakdown of the reasons for herbal medicine usage, indicating the number and percentage of respondents utilizing specific herbs for digestive health, cardiovascular well-being, nervous system support, diabetes management, mood and temperament, blood lipid control, general health, musculoskeletal health, respiratory well-being, immune system support, and other health-related reasons.

**Table 5:** Detailed breakdown of the reasons for medicinal herbs usage among CAM users.

Medicinal herbs		Reasons for use										
		Digestive	Cardiovascular	Nervous	Diabetes	Temperament	Dyslipidemia	General	Musculoskeletal	Respiratory	Immune system	Others
Thyme	Number	59	32	13	60	14	109	21	4	4	9	7
	Percentage	17.8%	9.6%	3.9%	18.1	4.2%	32.8%	6.3%	1.2%	1.2%	2.7%	2.1%
Chamomile	Number	1	0	154	6	12	2	2	6	1	0	4
	Percentage	0.5%	0.0%	81.9%	3.2%	6.4%	1.1%	1.1%	3.2%	0.5%	0.0%	2.1%
Green tea	Number	1	11	3	12	0	12	6	1	0	0	10
	Percentage	1.8%	19.6%	5.4%	21.4%	0.0%	21.4%	10.7%	1.8%	0.0%	0.0%	17.9%
Cinnamon	Number	0	4	8	169	15	6	13	8	0	0	7
	Percentage	0.0%	1.7%	3.5%	73.5%	6.5%	2.6%	5.7%	3.5%	0.0%	0.0%	3.0%
Ginger	Number	12	2	8	36	45	11	27	37	0	1	4
	Percentage	6.6%	1.1%	4.4%	19.7%	24.6%	6.0%	14.8%	20.2%	0.0%	0.5%	2.2%
Borage	Number	6	5	102	5	8	0	2	9	4	0	0
	Percentage	4.3%	3.5%	72.3%	3.5%	5.7%	0.0%	1.4%	6.4%	2.8%	0.0%	0.0%
Overall	Number	79	54	288	288	94	140	71	65	9	10	32
	Percentage	7.0%	4.8%	25.5%	25.5%	8.3%	12.4%	6.3%	5.8%	0.8%	0.9%	2.8%

### Associated Factors in CAM Use

Participants’ attitudes toward complications of CAM, side effects of routine drugs, effects of simultaneous consumption of CAM and routine drugs, and costs of CAM were assessed as potential associated factors for the use of CAM. The majority of participants (72.3%) believed that CAM has no side effects, while 27% believed that routine drugs have no side effects. Additionally, 63.3% of participants believed that concomitant use of routine drugs and CAM has a synergic effect. More than 95% of participants had previous positive experiences with CAM, and over 77% of patients considered the cost of CAM to be affordable. Logistic regression analysis revealed that the most significant associated factor was participants’ attitudes toward the side effects of CAM, and the concomitant use of routine drugs and CAM, as well as their previous positive experience with CAM (*P* < 0.001) (Table 6).

**Table 6:** Association between CAM use and patients’ attitudes and experiences among participants.

		CAM use				*P*-value	Odds ratio	95% Confidence intervals	
		Nonusers (n = 40)		Users (n = 336)					
		Count	%	Count	%			Lower	Upper
Patients’ attitude about side effects of CAM	No idea	22	8.1%	250	91.9%				
	Low	3	6.7%	42	93.3%		1.232	0.353	4.299
	Moderate	11	25.6%	32	74.4%	<0.001	0.256	0.114	0.577
	High	4	25.0%	12	75.0%		0.264	0.079	0.888
Patients’ attitude about side effects of routine drugs	No idea	13	12.7%	89	87.3%				
	Low	4	14.8%	23	85.2%		0.840	0.250	2.819
	Moderate	3	6.7%	42	93.3%	0.715	2.045	0.553	7.563
	High	20	9.9%	182	90.1%		1.329	0.632	2.794
Patient's view about concomitant use of routine drugs and CAM	Synergic effect	10	4.2%	228	95.8%				
	Interaction	8	16.0%	42	84.0%	<0.001	0.230	0.086	0.617
	None	22	25.0%	66	75.0%		0.132	0.059	0.292
Previous positive experience with CAM	Yes	6	37.5%	10	62.5%				
	No	34	9.4%	326	90.6%	<0.001	5.753	1.969	16.805
Previous negative experience with CAM	Yes	7	22.6%	24	77.4%				
	No	33	9.6%	312	90.4%	0.024	0.363	0.145	0.906
Patient's view on the costs of CAM	Inexpensive	14	9.0%	141	91.0%				
	Relatively inexpensive	6	11.1%	48	88.9%		0.794	0.289	2.183
	Acceptable	11	13.4%	71	86.6%	0.846	0.641	0.277	1.484
	Expensive	3	13.6%	19	86.4%		0.629	0.165	2.391
	So expensive	6	9.5%	57	90.5%		0.943	0.345	2.576

CAM = complementary and alternative medicine.

## Discussion

This cross-sectional study examined the prevalence of CAM use, particularly herbal remedies, and its associated factors among diabetic patients receiving healthcare at FUMS endocrinology clinics in southern Iran. The study found a high prevalence of CAM use, with more than 89% of patients reporting CAM use within the past year. Herbal preparations were the most commonly used type of CAM (99.4%). The study also revealed a significant association between CAM use and patients’ psychological health, indicating that individuals with higher levels of psychological well-being were more likely to use CAM. Patient attitudes toward the safety of CAM, the synergistic effects of concomitant use of routine drugs and CAM, and previous positive experiences with CAM were identified as important factors influencing CAM use.

Several demographic factors were explored to understand the patterns of CAM use. Notably, a higher prevalence of CAM use was observed among married individuals and women. Education levels did not show a significant difference in the use of CAM, contrary to a study in India where education and socio-economic status were strongly correlated with complementary medicine use.^28^ The lack of significant differences in CAM use based on residence, insulin therapy, and oral therapy suggests a widespread adoption of CAM across different subgroups of diabetic patients.

Comparing the prevalence of CAM use in different countries, this study's findings align with reports from Africa, Asia, Australia, and Europe, which have shown CAM use prevalence ranging from 50% to 90%.^29^, ^30^, ^31^, ^32^, ^33^ In Iran, previous studies have reported CAM use prevalence ranging from 10% to 89.22% in different target populations.^34^, ^35^, ^36^, ^37^ The accessibility, affordability, and perceived safety of CAM, along with concerns about the adverse effects of conventional medications, contribute to its widespread use.^38^^,^^39^ Moreover, the long-standing history of CAM use in different cultures and the increasing availability of health information have also contributed to its popularity. However, despite its widespread use, CAM has not received official recognition in many countries due to the lack of convincing safety and efficacy data. The variability in prevalence can be attributed to differences in study populations, periods, and sample selection. Notably, this study reported a higher prevalence of CAM use among diabetic patients (89%) compared to a previous study conducted in Shiraz, Iran (75.3%).^11^^,^^12^^,^^40^^,^^41^ This difference may be due to the inclusion of patients from smaller cities and rural areas, where CAM use tends to be more prevalent. The observed increase in CAM use prevalence over time aligns with trends observed in national surveys conducted in various countries.^1^^,^^42^^,^^43^ The factors associated with CAM use among diabetic patients in this study included patients’ psychological health, attitude toward the safety of CAM, belief in the synergistic effects of combining routine medications with CAM, and previous positive experiences with CAM. These findings suggest that patients’ attitudes, beliefs, and experiences play a significant role in their decision to use CAM. Healthcare providers should be aware of these factors and engage in open discussions with patients to better understand their preferences and provide appropriate guidance.

Consistent with previous studies in Iran, herbal preparations were the most commonly used type of CAM in this study.^11^^,^^44^^,^^45^ This finding indicates a preference for herbal remedies among Iranian patients, possibly due to unfamiliarity with other complementary therapies. The inclusion of objective patient characteristics such as HbA1c in this study strengthens the understanding of the study population and contributes to its homogeneity.

The investigation into clinical parameters revealed intriguing associations. While HbA1c levels in individuals using CAM were found to be lower than in others, this difference did not reach statistical significance. The lack of a significant impact of CAMs on height and weight raises questions about the specific therapeutic effects of these modalities on diabetes-related outcomes. The study contributes to the existing literature by providing insights into the diverse factors influencing CAM use in the context of diabetes management.

An alarming observation is that approximately 80% of individuals did not consult their physicians about using CAM. This lack of communication can have potentially serious consequences, considering the complexity of diabetes management and the need for coordinated care. The prevalence of CAM use for various purposes, including blood sugar control, blood fat reduction, and stress relief, underscores the multifaceted roles attributed to these alternative approaches by diabetic patients.

The positive views of patients regarding the reasonable cost of various CAM treatments and the belief in the harmlessness of CAM further emphasize the need for open discussions between healthcare professionals and patients about CAM practices. The findings align with a study, which reported that only 13% of individuals informed their physicians about using complementary medicine.^46^ However, the disclosure rate appears to be increasing, as evidenced by the study by Mousavi and Mahmoudian^47^, where about 54% of participants disclosed their use of CAM to their treating physicians.^47^

Comparative analysis with studies in other regions, such as Palestine and Jordan, highlights varying disclosure rates and satisfaction levels among diabetic patients using CAM.^48^^,^^49^ The study suggests that the increasing prevalence of CAM in societies like Fasa may be attributed to historical practices, the integration of complementary methods from various regions, and overall satisfaction with the effects of these alternative treatments.

The study also highlighted the need for healthcare professionals to be knowledgeable about CAM modalities and their potential interactions with conventional diabetes medications. Only a small percentage of patients (20.8%) in this study informed their physicians about their use of herbal drugs. This lack of communication can lead to potential risks and adverse effects, as certain herbal preparations may interact with diabetes medications and affect blood glucose control. Therefore, healthcare providers should proactively inquire about CAM use during patient consultations and create a supportive environment for patients to discuss their CAM practices openly. Understanding the factors influencing CAM use among diabetic patients can inform healthcare providers and policymakers in developing appropriate strategies for integrating CAM approaches into conventional diabetes care. It is important to establish evidence-based guidelines for the safe and effective use of CAM in diabetes management. Furthermore, healthcare providers should receive education and training on CAM modalities to enhance their knowledge and ability to provide informed guidance to patients.

Interestingly, a considerable percentage of patients believed in the high side effects of chemical drugs, emphasizing the need for increased awareness and education. The study reveals a significant belief among users of CAM in enhancing the effects of chemical drugs by using them simultaneously, aligning with findings from a study in Shiraz.^12^ This dual-use approach may have implications for the overall efficacy and safety of diabetes management strategies.

An important aspect addressed in this study is the correlation between CAM use and QoL. While previous studies have yielded mixed results, this study's findings add to the literature by demonstrating that CAM use is not significantly associated with QoL in diabetic patients.^50^, ^51^, ^52^, ^53^, ^54^ It should be noted that different methods were employed in measuring QoL across studies, which may contribute to the variability in results. The present study's findings suggest that intensive use of CAM practitioners may be associated with lower QoL in patients with T2DM and/or cardiovascular disease.

The results of our study shed light on the utilization of various herbal plants among individuals with diabetes. We observed that patients not only turned to herbal medicine for blood sugar control but also sought improvements in other health aspects, such as blood lipid levels, nervous system health, cardiovascular health, and overall well-being. This highlights the holistic approach that individuals with diabetes adopt in managing their condition, addressing multiple dimensions of health simultaneously. The extensive diversity of herbal medicines reported by the participants underscores the wide range of options available in traditional medicine. While we have only highlighted a few of the most prevalent herbal plants, it is important to note that the usage of other plants was reported by a smaller number of individuals, limiting our ability to draw robust conclusions regarding their efficacy.

The prevalence of various medicinal plants, with thyme, cinnamon, chamomile, ginger, yarrow, and green tea being the most commonly used, highlights the rich landscape of herbal medicine in Fasa. The observed high mental health scores in users of herbal plants, especially those with nerve-calming and anxiety-reducing properties, suggest potential psychosocial benefits associated with herbal remedies.

The utilization of CAM among individuals managing T2DM is a complex and multifaceted phenomenon. Our study sought to explore factors associated with CAM usage within the Iranian population. Several studies have delved into the determinants influencing CAM utilization among individuals with DM, shedding light on various sociodemographic and clinical factors.^55^^,^^56^ Our findings align with previous research, indicating that factors such as the type of disease, sex, marital status, education, and residency can significantly influence the choice to incorporate CAM into the diabetes management regimen.

Surprisingly, despite the prevalence of CAM use in our study, our results did not reveal a significant impact, either positive or negative, of CAM on glucose and lipid control variables. The intricate interplay between CAM modalities and conventional pharmacotherapy is an area of ongoing research.^57^ However, it is crucial to interpret our results cautiously, considering the limitations of our retrospective study design and the inherent challenges in establishing causal relationships.^58^

Furthermore, the potential interaction of CAM with conventional medications is a subject of increasing importance. While some studies suggest possible interactions that may influence treatment outcomes,^57^ the evidence remains inconclusive, warranting further investigation into the specific herb-drug interactions and their clinical implications.^59^ Clinicians should remain vigilant and engage in open communication with patients about their use of CAM to ensure comprehensive and coordinated diabetes management.^58^

A distinctive feature of our research lies in the comprehensive exploration of CAM, with a specific emphasis on herbal preparations. The high prevalence of CAM use, particularly herbal remedies, observed in our study population sheds light on the prominence of traditional healing practices in the management of diabetes.

Moreover, our study contributes novel findings regarding the association between CAM use and patients’ psychological health, attitudes toward safety, beliefs in synergistic effects, and prior positive experiences with CAM. These factors provide a nuanced understanding of the motivations behind CAM utilization among diabetic individuals, offering healthcare providers and policymakers valuable insights into patient perspectives.

### Limitations

This study has several limitations that should be considered. Firstly, the use of a clinic-based sample introduces a potential selection bias, as the population attending endocrinology clinics may differ from those not seeking specialized care. While this approach facilitated targeted sampling of diabetic patients, caution should be exercised in generalizing the findings to the broader population. Future research encompassing a more diverse participant pool, including those not attending specialized clinics, is warranted to enhance the external validity of the results. Secondly, the study used a convenience sample, which may limit the generalizability of the findings to the entire diabetic population in Fasa. Thirdly, the study relied on self-reported data, which may be subject to recall bias and social desirability bias. Also, the study did not assess the specific efficacy or safety of the CAM modalities used by the participants. Future research should aim to address these limitations and explore the specific effects of different CAM modalities on diabetes outcomes.

## Conclusion

In conclusion, this study unveils the prevalence and determinants of CAM use among diabetic patients attending endocrinology clinics in southern Iran. Our findings emphasize the significance of psychological health, attitudes towards CAM safety, beliefs in synergistic effects with routine medications, and positive prior experiences as associated factors influencing CAM utilization. Recognizing these determinants is crucial for healthcare providers to engage in patient-centered discussions, fostering an open dialogue about CAM practices, and ensuring comprehensive diabetes care. Further research is warranted to delve into the specific modalities of CAM used, their efficacy, and potential interactions, contributing to evidence-based guidelines for integrated diabetes management.

## Declaration of competing interest

The authors declare that they have no known competing financial interests or personal relationships that could have appeared to influence the work reported in this paper.
